# Phase Transformations from Nanocrystalline to Amorphous (Zr_70_Ni_25_Al_5_)_100-x_W_x_ (x; 0, 2, 10, 20, 35 at. %) and Subsequent Consolidation

**DOI:** 10.3390/nano11112952

**Published:** 2021-11-03

**Authors:** M. Sherif El-Eskandarany, Naser Ali, Fahad Al-Ajmi, Mohammad Banyan

**Affiliations:** Nanotechnology and Applications Program, Energy and Building Research Center, Kuwait Institute for Scientific Research, Safat 13109, Kuwait; nmali@kisr.edu.kw (N.A.); ftajmi@kisr.edu.kw (F.A.-A.); mbanyan@kisr.edu.kw (M.B.)

**Keywords:** metallic glasses, mechanical alloying, cold-rolling, plastic deformation, glass forming ability, crystal structure, morphology, crystallization, powder consolidation, microhardness

## Abstract

Glasses, which date back to about 2500 BC, originated in Mesopotamia and were later brought to Egypt in approximately 1450 BC. In contrast to the long-range order materials (crystalline materials), the atoms and molecules of glasses, which are noncrystalline materials (short-range order) are not organized in a definite lattice pattern. Metallic glassy materials with amorphous structure, which are rather new members of the advanced materials family, were discovered in 1960. Due to their amorphous structure, metallic glassy alloys, particularly in the supercooled liquid region, behave differently when compared with crystalline alloys. They reveal unique and unusual mechanical, physical, and chemical characteristics that make them desirable materials for many advanced applications. Although metallic glasses can be produced using different techniques, many of these methods cannot be utilized to produce amorphous alloys when the system has high-melting temperature alloys (above 1500 °C) and/or is immiscible. As a result, such constraints may limit the ability to fabricate high-thermal stable metallic glassy families. The purpose of this research is to fabricate metallic glassy (Zr_70_Ni_25_Al_5_)_100-x_W_x_ (x; 0, 2, 10, 20, and 35 at. %) by cold rolling the constituent powders and then mechanically alloying them in a high-energy ball mill. The as-prepared metallic glassy powders demonstrated high-thermal stability and glass forming ability, as evidenced by a broad supercooled liquid region and a high crystallization temperature. The glassy powders were then consolidated into full-dense bulk metallic glasses using a spark plasma sintering technique. This consolidation method did not result in the crystallization of the materials, as the consolidated buttons retained their short-range order fashion. Additionally, the current work demonstrated the capability of fabricating very large bulk metallic glassy buttons with diameters ranging from 20 to 50 mm. The results indicated that the microhardness of the synthesized metallic glassy alloys increased as the W concentration increased. As far as the authors are aware, this is the first time this metallic glassy system has been reported.

## 1. Introduction

Metallic materials are crystalline in nature, with translational symmetry; that is, their constituent atoms are arranged in the space, with a regular and periodic fashion [1]. The classic concept of long-range atomic arrangement metals revolutionized in 1960 when Duwez and his team synthesized an Au–25 at. % Si alloy in the glassy state by rapidly solidifying the melt with an extraordinary cooling rate approaching a million degrees per second [2]. The x-ray diffraction (XRD) pattern of the as-rapidly soldificated Au_75_Si_25_ alloy revealed no crystalline peaks. However, it demonstrated a couple of broad and diffuse peaks. This implied the presence of a noncrystalline structure and formation of amorphous phase.

Since the first pioneer investigation for preparing amorphous metallic alloys, almost all amorphous materials have been synthesized exclusively by rapid solidification of melts or vapors [3], atomic disordering of crystalline lattices [4], solid-state amorphization reaction between pure elements [5], and solid-state transformations from metastable phases [6]. Additionally and very importantly, Yeh et al. [7] discovered the dissolution of hydrogen gas in crystalline Zr_3_Rh results in amorphous Zr_3_RhH_5.5_. It was demonstrated that chemical energies could be used to drive a solid-state crystal-to-amorphous transformation. Because hydrogen atoms are small, they can easily diffuse in crystalline intermetallics of large unit cells, allowing the reaction to take place at temperatures lower than the amorphous hydride’s crystallization temperature. This method has so far been used to produce a variety of amorphous alloys [8,9]. Herd et al. [5] reported yet another type of solid-state amorphization reaction when they discovered that metals, such as tellurium, selenium, and silicon, can diffuse into amorphous semiconductors at low temperatures.

In 1983, Schwarz and Johnson demonstrated the first reaction of two pure crystalline metals to form a single phase of an amorphous alloy [10]. Thin films of pure gold and lanthanum a few tenths of a nanometer thick were fully reacted at 343 K in a matter of kiloseconds [10]. Rutherford back-scattering spectroscopic (RBS) marker experiments on a Ni/Zr diffusion couple [6] and transmission electron microscopy (TEM) on Co/Zr were used to investigate the micro-mechanism of the solid-state amorphizing reaction in multilayers. These experiments show that the moving species is the smaller atom in the diffusion couple. The diffusion coefficient through this amorphous layer determined the growth rate of the amorphous layer formed at the interface of the two atomic species. Recently, a significant number of new metallic glassy alloys have been discovered that have the potential to be used in wide range of applications. Of these, Zr-, Ti-, Mg, Cu-, Ni, Cr, Co, and Fe-based systems can be used for biomedical [11,12,13,14,15,16], structural [17,18,19,20,21,22,23,24,25,26,27,28], magnetic [29,30,31,32], optical and electronical [33,34,35], anticorrosion/erosion protective coating [36,37,38], and catalytic [39,40,41,42,43,44,45,46] applications. Several useful publications are available that include details on the fabrication techniques, characterizations, and implementations of a wide range of metallic glassy alloys (see, for example, Refs. [47,48,49,50,51,52,53,54]).

Apart from the conventional methods for preparing amorphous and metallic glassy alloys, Koch et al. [55] proposed a new technique called mechanical alloying (MA) for synthesizing amorphous materials far below the crystallization temperature by high-energy ball milling an elemental mixture of Ni_60_Nb_40_ powders for approximately 40 ks in an air or a helium atmosphere. Throughout the 1980s, and in part to better understand the mechanism of amorphous formation via MA, a variety of simple amorphous systems [56], such as Ni-Ti [57,58], Ni-Zr [59], and Fe-Zr [60], were synthesized via MA, where their properties compared to those obtained via traditional technique. It has been established that the MA process is a typical mechanically driven solid-state reaction that occurs between freshly formed layers of reactant metallic powders (diffusion couples) at ambient temperature. The results from various metallic systems reveal that MA is capable of fabricating homogeneous amorphous with a wider glass-forming ability (GFA) that can cover roughly 80% of the composition range for a binary system. The consistent success of preparing various amorphous systems resulted in the addition of MA technique as a desirable and powerful method for the production of amorphous and metallic glassy alloys, particularly for those systems that cannot be prepared by conventional methods [56].

Extraordinary high melting point alloys, such as Al-Ta [61], AlNb [62], and AlHf [63], as well as immiscible systems (e.g., Cu-Ta [64]), are some of those difficult alloys and systems that can be obtained by the MA process. Within the last two decades, various high-temperature multicomponent metallic glassy systems, such as Nb_50_Zr_10_Al_10_Ni_10_Cu_20_ [65], V_45_Zr_20_Ni_20_Cu_10_Al_2.5_Pd_2.5_ [66], Ta_55_Zr_10_Ni_10_Al_10_Cu_15_ [67], and Zr_65_Al_7.5_Ni_10_Cu_12.5_Pd_5_ [68], were successfully prepared by the MA approach. These metallic glassy systems revealed excellent thermal stability with supercooled liquid regions (ΔT_x_) in the range between 80 and 110 °C. The existence of such a wide ΔT_x_ has prompted researchers to use temperature-controlled hot pressing, as well as spark plasma sintering procedures to consolidate the glassy powders into full-dense bulk metallic glassy buttons [69,70,71,72].

Due to the high melting point of tungsten (W/3422 °C), homogeneous amorphous/metallic glassy alloys are extremely difficult to fabricate using standard processes, such as melt spinning (MS) or the melting/casting process. As a result of the high melting temperature differences between W and the majority of pure metals, homogeneous W-based and/or W-rich metallic glassy systems are difficult to fabricate. The first successful example for fabrication of an equiatomic FeW amorphous alloy was reported in 1997, when El-Eskandarany et al. used a typical MA approach to fabricate a homogeneous amorphous phase using a low-energy ball mill [73]. Since then, W has attracted many researchers to use it as an alloying element (~2–5 at. %) for fabricating high-thermal stable amorphous/metallic glassy alloys. However, multicomponent Gd_x_Zr_10_Fe_58-x_Co_10_B_15_Mo_5_W_2_ (where: x = 0, 1, 2, 3, 4, 5) metallic glassy alloys were synthesized through the MS technique [74]. Due to the low-W concentration (2 at. %), the metallic glassy phase was successfully formed.

Herein we report the influence of W additions at concentrations ranging from 0 to 35% at. % on the glass forming ability (GFA) and subsequent crystallization of the metallic glassy Zr_70_Ni_25_Al_5_ ternary system. Additionally, and to the authors’ knowledge, the effect of pre-mechanical treatment via cold rolling (CR) of the feedstock powders (Zr_70_Ni_25_Al_5_)_100-x_W_x_ (x; 0, 2, 10, 20, 35 at. %) prior to high-energy ball milling was studied. To investigate the influence of W additives on the bulk density and microhardness of metallic glassy systems, the as -CR/MA powders were consolidated into bulk metallic glassy buttons using the SPS approach. Finally, the present work demonstrates a systematic study of a hitherto unreported metallic glassy system.

## 2. Materials and Methods

### 2.1. Feedstock Materials

Pure (99.5 wt.%) elemental powders of Zr (50 μm), Ni (45 μm), Al (10 μm), and W (10 μm), purchased from Sigma–Aldrich, Inc., St. Louis, MO 68178, USA, were used as precursor materials. The starting powders of Zr, Ni, and Al were blended inside a helium (He_ glove box (mBRAUN, Glove Box Workstation UNILAB Pro, Dieselstr. 31, D-85748 Garching, Germany)) to give six patches with nominal compositions (at. %) of Zr_70_Ni_25_Al_5_ and (Zr_70_Ni_25_Al_5_)_100-x_W_x_(x; 2, 5, 10, 20, 35 at. %). The patch weighed approximately 50 g.

### 2.2. Sample Preparations

#### 2.2.1. Zr_70_Ni_25_Al_5_ Ternary System

The Zr_70_Ni_25_Al_5_ powders mix was handled within the glove box and then charged into a tool steel vial (200 mL capacity) supplied by evico GmbH, Großenhainer Str. 101, 01,127 Dresden, Germany, together with 60 tool steel balls (10 mm in diameter) at a 45:1 ball-to-powder weight ratio. The vial was then loaded on a high-energy ball mill (PM 100), supplied by Retsch GmbH, Retsch–Allee 1–5, 42,781 Haan, Germany, and rotated at a speed of 250 rpm for 1, 6, 12.5, and 25 h.

#### 2.2.2. Multicomponent (Zr_70_Ni_25_Al_5_)_100-x_W_x_(x; 2, 5, 10, 20, 35 at. %) Systems

To ensure homogeneity of the mix, the powders from each patch were first charged into a 200 mm-long, 0.5 mm-diameter stainless steel (SUS 304) tube and then sealed in the glove box under He atmosphere. Each patch’s sealed tube was manually cold rolled 100 times using a two-drum cold rolling machine. The cold-rolled systems were then opened inside the glove box, and the discharged powders were placed into milling vials using the same experimental milling settings as previously described for the Zr_70_Ni_25_Al_5_ ternary system. The powders in these systems were milled at a speed of 250 rpm for 25, 50, and 100 h.

### 2.3. Powder Consolidation

The powders obtained after ball milling were individually consolidated into dense buttons through spark plasma sintering (SPS), acquired from Dr. Sinter Lab. Instrument Co., Kagaku Analys AB Johanneberg Science Park, Sven Hultins gata 9 B, 412 58 Göteborg, Sweden. The system is comprised of a press with vertical single-axis pressurization, electrodes incorporating a water cooler, a water-cooled vacuum chamber, a vacuum/air/argon-gas atmosphere control mechanism, a special DC (direct current) pulse sintering power generator, a cooling-water control unit, a *Z*-axis position and control unit, temperature position and control units, and applied pressure dummies. The powders obtained in this study were put onto a graphite die. Additionally, graphite sheets were utilized to prevent interactions between surfaces. To minimize heat transmission, the die was wrapped with carbon felt and secured with a carbon yard. Control of the sintering process was accomplished via the application of an electric field. We utilized sintering SPS in this study, which involves internally heating samples through electric current flow. Heating and cooling rates of 580 and 280 K/min were utilized, respectively. External pressure in the range of 10–15 MPa was applied during sintering. The whole procedure took approximately 6 min. Additional information on this SPS experiment procedure for other systems has been published elsewhere [75].

### 2.4. Sample Characterizations

#### 2.4.1. Crystal Structure

X-ray diffraction (XRD) examination was performed utilizing a SmartLab–Rigaku (Rigaku Corporation, Tokyo, Japan) XRD with CuKα radiation equipment at a power output of 9 kW. Field-emission high-resolution transmission electron microscopy (HRTEM, JOEL-2100F, Tokyo, Japan) was also utilized in conjunction with scanning transmission electron microscopy (STEM) through an Oxford Instruments energy dispersive spectroscopy (EDS, Asylum Research, NanoAnalysis, 25.2 mi, High Wycombe, UK) outfitted with a JEOL-2100F. This microscope’s objective lens has a spherical aberration coefficient (Cs) of 0.5 mm, a point resolution of 0.19 nm, and a lattice resolution of 0.12 nm. The nano-beam diffraction (NBD) spot sizes were 0.5 and 25 nm. Additionally, the TEM specimens were prepared as a consolidated sample using a Cryo Ion IB-09060CIS Slicer machine (JOEL-2100F, Tokyo, Japan).

#### 2.4.2. Morphology

The samples were studied using field-emission scanning electron microscopy (FE-SEM) at a 15 kV voltage (JSM-7800F JEOL Co. Tokyo, Japan) and elemental analysis using an Oxford Co. EDS interface.

#### 2.4.3. Thermal Analysis

The glass-forming ability indexed by glass transition temperature, thermal stability indexed by crystallization temperatures, and melting temperature were investigated through high-temperature differential scanning calorimetry (HT-DSC, LABSYS evo DSC/T_g_/DTA), supplied by Setaram Instrumentation, Seine-Port, France).

#### 2.4.4. Density and Microhardness

Archimedes’ principle was used to measure the density using toluene medium. The microhardness of compacted samples was determined using a 500 g Vickers indenter with an average reading of ten indentations.

## 3. Results and Discussions

### 3.1. Changes in Structure, Morphology, and Composition Associated with Changing the MA Time

#### 3.1.1. Metallic Glassy Zr_70_Ni_25_Al_5_ System

**Figure 1:**
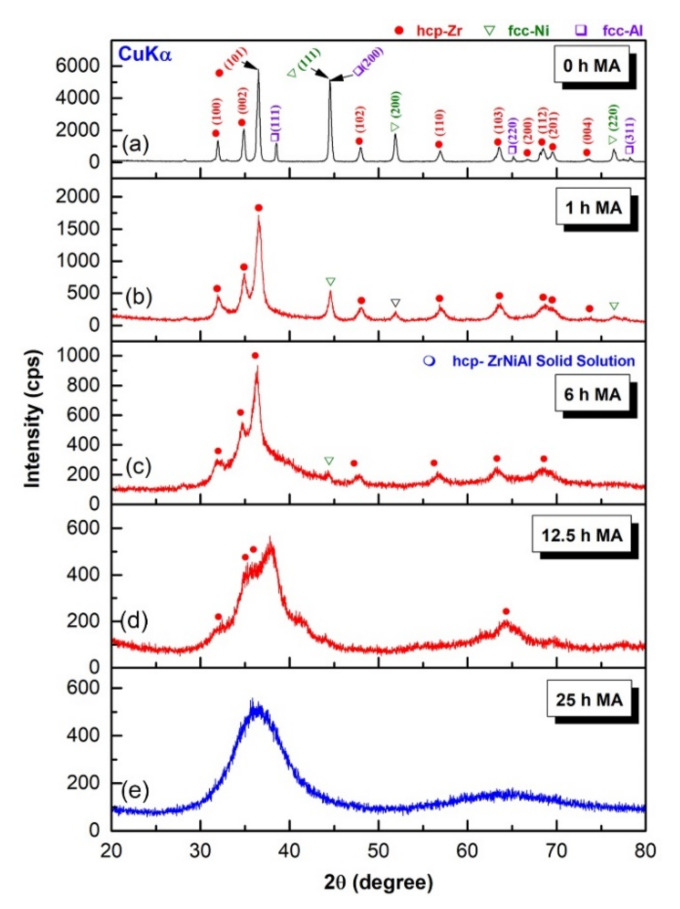
XRD patterns of MA-Zr_75_Ni_25_Al_5_ powders obtained after HEBM for (**a**) 0, (**b**) 1, (**c**) 6, (**d**) 12.5, and (**e**) 25 h.

**Figure 2:**
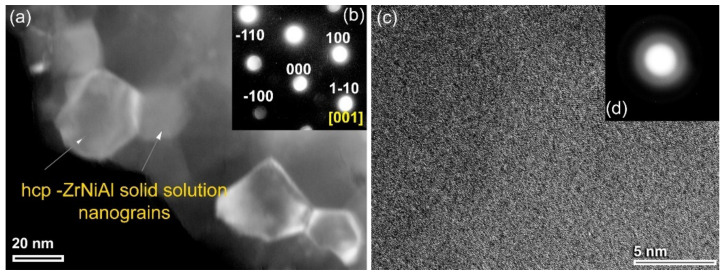
The field-emission high-resolution transmission electron microscope (FE-HRTEM) images and corresponding nanobeam diffraction patterns (NBDPs) of MA-Zr_75_Ni_25_Al_5_ powders milled for 6 h and 25 h are displayed in (**a**,**b**) and (**c**,**d**), respectively.

We shall begin by presenting the structural changes to the MA base material of elemental Zr_70_Ni_25_Al_5_ powders for various stages of high-energy ball milling (HEBM). The x-ray diffraction pattern (XRD) of the starting elemental powders is displayed in Figure 1a. The sample revealed sharp Bragg peaks, which correspond to crystalline hcp -Zr, fcc- Ni, and fcc -Al (Figure 1a). At the early stage of MA (1–6 h), the Bragg peaks related to fcc -Al and -Ni tended to diffuse into the hcp -Zr lattice to form a partial hcp -ZrNiAl solid solution phase coexisting with unprocessed Ni crystals, as shown in Figure 1b. Continuous HEBM (6 h) led to enhance the mechanically driven solid-state diffusion, resulting in a decrease in the volume fraction of free Ni crystals, as evidenced by the disappearance of Ni(200) and Ni(220) (Figure 1c). The evident broadening of the Bragg peaks for hcp -ZrNiAl (101) indicates the formation of nanocrystalline powders (~36 nm) (Figure 1c), as confirmed by a field-emission high-resolution transmission electron microscope (FE-HRTEM) image (Figure 2a). The local crystal structure of a single equiaxed grain for this sample was examined and determined to be hcp -ZrNiAl using nano beam diffraction (NBD) orientated [001], as indexed in Figure 2b.

Because hcp -ZrAlNi solid solution is a metastable phase, it is extremely susceptible to mechanically induced deformation, resulting in lattice defects with HEBM for additional MA time (12.5 h). Accordingly, a solid-state vitrification process was realized upon mechanically stimulating the hcp–solid solution atoms to a metastable amorphous solid state. This solid-state phase transformation was evidenced by development of a broad diffuse peak following the middle stage of MA (12.5 h), which coexisted with a minor volume fraction of untransformed hcp -ZrNiAl nanocrystals (Figure 1d). After 25 h of MA (final stage), the XRD pattern of the sample exhibited two haloes centered at approximately 36° and 64°, where the Bragg peaks belonging to the untransformed hcp -solid solution phase had vanished, as displayed in Figure 1e.

The FE-HRTEM technique was employed to confirm the completion of the vitrification process and formation of amorphous structure. The atomic resolution TEM image (Figure 2c), displayed in conjunction with the NBDP (Figure 2d), demonstrated that the sample exhibited a maze pattern contrast without evidence of any crystalline phase precipitation (s). Moreover, the NBDP taken from the middle part of the image displays a typical halo-diffraction of an amorphous phase, as elucidated in Figure 2d.

#### 3.1.2. Metallic Glassy (Zr_70_Ni_25_Al_5_)_100-x_W_x_ (x; 0, 2, 10, 20, 35 at. %) Systems

**Figure 3:**
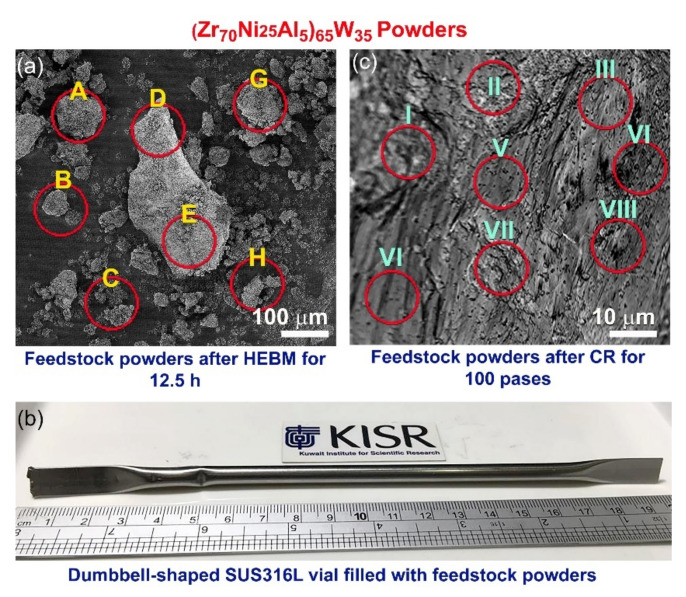
(**a**) A micrograph of the field-emission scanning electron microscope (FE-SEM) for the powders obtained after 12 h MA, (**b**) a photo of a dumbbell-shaped SUS304 vial filled with the powders and sealed under He gas atmosphere prior to the CR process, and (**c**) an FE-SEM micrograph of the powders after CR for 100 passes. The red circular symbols displayed in (**a**,**c**) refer to the zones selected for local elemental analysis via SEM/energy-dispersive X-ray (EDS) spectroscopy. The corresponding EDS results of the zones A-H and I-VIII are listed in Table 1

In accordance with the study’s objectives, the effect of the W-refractory alloying element on the thermal stability and microhardness of metallic glassy Zr_70_Ni_25_Al_5_ powders has been investigated as a function of W content (x). The field-emission scanning electron microscope (FE-SEM) image of (Zr_70_Ni_25_Al_5_)_65_W_35_ powders obtained after 12.5 h of MA is displayed in Figure 3a. Because of the cold-welding effects caused by HEBM, the powders were agglomerated to form irregularly shaped large particles ranging in size from a few μm to 650 μm, as shown in Figure 3a. More seriously, the energy-dispersive X-ray spectroscopy (EDS)/SEM analysis indicated that the local composition of the HEBM powders obtained after this step of MA differed substantially from particle to particle and within individual ones, as shown in Table 1.

**Figure 4:**
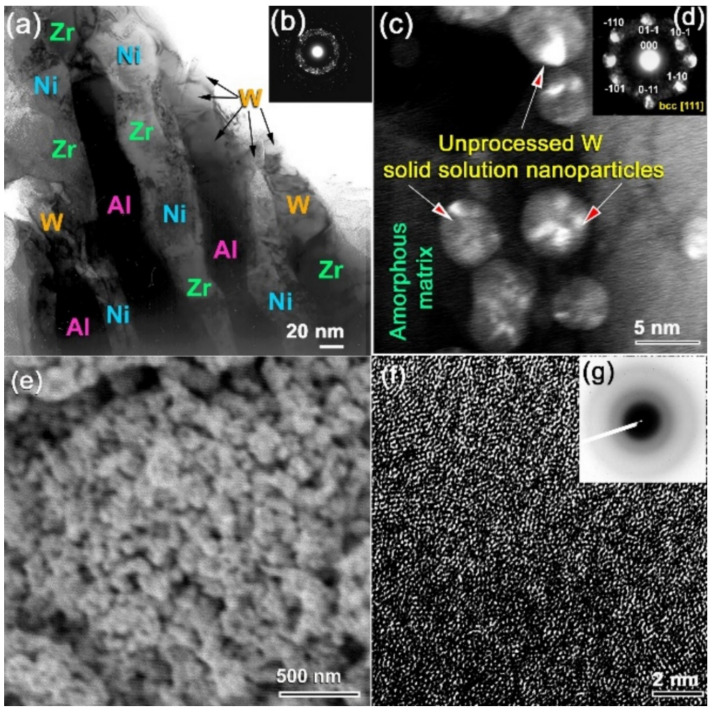
(**a**,**b**), and (**c**,**d**) show the FE-HRTEM images, and corresponding selected area diffraction patterns (SADPs) of the powders obtained after CR for 100 passes and CR for 100 passes followed by 25 h MA, respectively. (**e**) FE-SEM image of the powders CR for milling for 50 h after 100 passes of CR. The FE-HRTEM image and its related nanobeam diffraction pattern (NBDP) are presented in (**f**,**g**), respectively.

In the present work, we proposed a crucial cold rolling (CR) step prior to the HEBM process. The multicomponent powders of the feedstock materials were carefully blended in a glove box under helium gas (He) atmosphere before being sealed into a dumbbell -shaped SUS304 can (Figure 3b). Then, the sealed can was severely CR for 50 and 100 passes, using a conventional CR machine. The purpose of utilizing the CR step was not only to produce homogeneous composite powders but also to subject the powder particles to severe plastic deformation, which can assist in the phase transformation procedure occurring during the HEBM process. After 25 passes of CR, the starting materials tended to form nanocomposites containing intimate multilayers of the metallic alloying, as shown in Figure 4a. The corresponding selected area diffraction pattern (SADP) implied the existence of polycrystalline phases related to the alloying elements of Zr, Ni, Al, and W without any evidence of the formation of an amorphous phase (Figure 4b). This is indicated by the lack of a halo-diffuse pattern and the existence of a continuous Dybe ring pattern coexisting with sharp spots of the elemental powders (Figure 4b).

**Figure 5:**
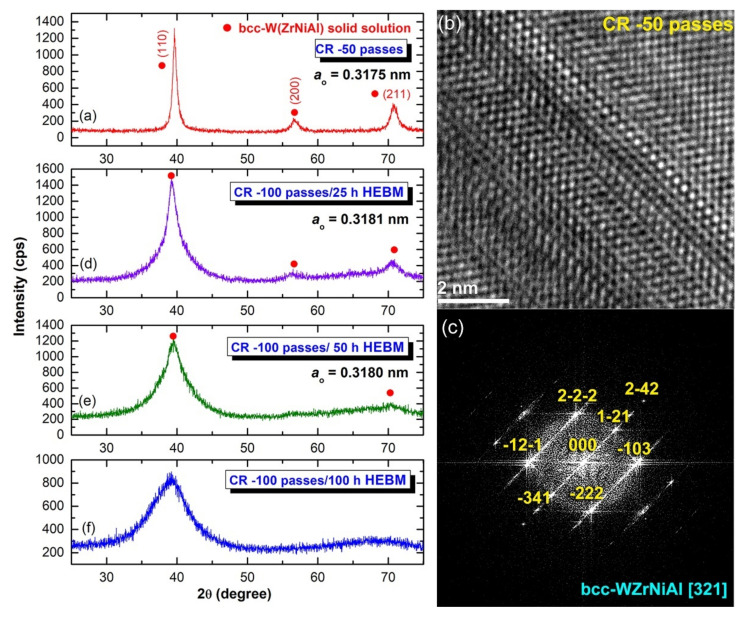
(**a**) XRD patterns of as-CR-(Zr_75_Ni_25_Al_5_)_65_W_35_ powders for 50 passes, (**b**) atomic resolution lattice image of 50 CR passes, and (**c**) Fast Fourier transform (FTT). The XRD patterns of as-CR-(Zr_75_Ni_25_Al_5_)_65_W_35_ powders for 100 passes and then HEBM for 25 h, 50 h, and 100 h are displayed in (**d**–**f**), respectively.

The XRD pattern of the sample CR for 50 passes revealed bcc -structure of metastable WZrNiAl solid solution phase, as shown in Figure 5a. This implies the CR’s capability to conduct a solid-state diffusion starting from elemental multicomponent metallic powders. Additionally, the as–CR (Zr_70_Ni_25_Al_5_)_65_W_35_ powders obtained after 50 passes revealed severe plastic deformation, as indicated by the presence of stacking faults and nano-twins in the W lattice (Figure 5b), which was orientated to [110], as shown in Figure 5c. The lattice parameter (a_o_) of this obtained solid solution phase was calculated and found to be 0.31752 nm, which is a bit larger than the reported value (0.31738 nm) (Figure 5a). More notably, the EDS analysis from different zones of the sample obtained after 50 passes of CR (Figure 3c) revealed a uniform composition close to the starting nominal composition with no significant differences, as indicated in Table 1.

The possibility of solid solution-to-amorphous phase transformation upon HEBM of CR 100 passes was investigated by milling the CR powders for 25 h. The XRD pattern of the sample obtained after 25 h of HEBM, which is displayed in Figure 5d revealed broad Bragg lines related to the bcc -WZrNiAl solid solution phase. The a_o_ of this sample was calculated and found to be 0.3181 nm, indicating an expansion conducted in the solid solution phase upon introducing lattice imperfections during HEBM (Figure 4d). The Bragg peaks, exemplified by W(ZrNiAl)–(110), showed a significant broadening after 50 h of HEBM; however, its a_o_ (0.3180 nm) did not show any further increase, as displayed in Figure 5e.

The FE-HRTEM analysis of this sample indicated that after 50 h of milling, a larger volume fraction of W(ZrNiAl) solid solution transformed into an amorphous phase, as implied by the fine amorphous matrix shown in Figure 4c. After this stage of HEBM, a considerable volume fraction of untransformed solid solution phase consisting of spherical nanoparticles coexisted with the amorphous matrix (Figure 4c,d). After the final stage of HEBM (100 h), all the Bragg peaks related to the nanocrystalline W(ZrNiAl) solid solution phase were replaced by diffuse halo peaks, indicating the formation of an amorphous phase of (Zr_70_Ni_25_Al_5_)_65_W_35_ powders (Figure 5f).

The final product of amorphous powders consisted of ultrafine particles with an average size of 120 nm, as shown in Figure 4e. Additionally, the FE-HRTEM and its corresponding NBDP of a 100 h sample confirmed the formation of amorphous phase, as shown in the maze-like morphology and the diffuse rings displayed in Figure 4f,g, respectively.

**Figure 6:**
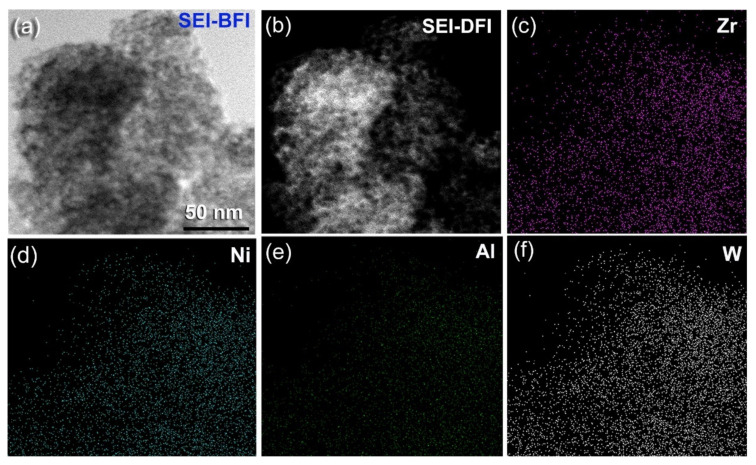
Local elemental analysis and structural characteristics of (Zr_75_Ni_25_Al_5_)_65_W_35_ powders obtained after 100 passes of CR and then HEBM for 100 h; (**a**) scanning transmission-bright field image (SEI-BF), (**b**) scanning transmission-dark field image (SEI-DF), and the corresponding EDS elemental mapping of (**c**) Zr, (**d**) Ni, (**e**) Al, and (**f**) W.

Additionally, the scanning transmission electron (SE) bright field image (BFI) and dark field image (DFI) of the sample obtained after 100 h of HEBM indicates the development of spherical nanopowders with an average diameter of 5 nm (Figure 6a,b). The related TEM/EDS X-ray mapping for Zr (Figure 6c), Ni (Figure 6d), Al (Figure 6e), and W (Figure 6f) depict the uniform distribution of the alloying elements, indicating the production of a chemically homogenous amorphous phase.

**Figure 7:**
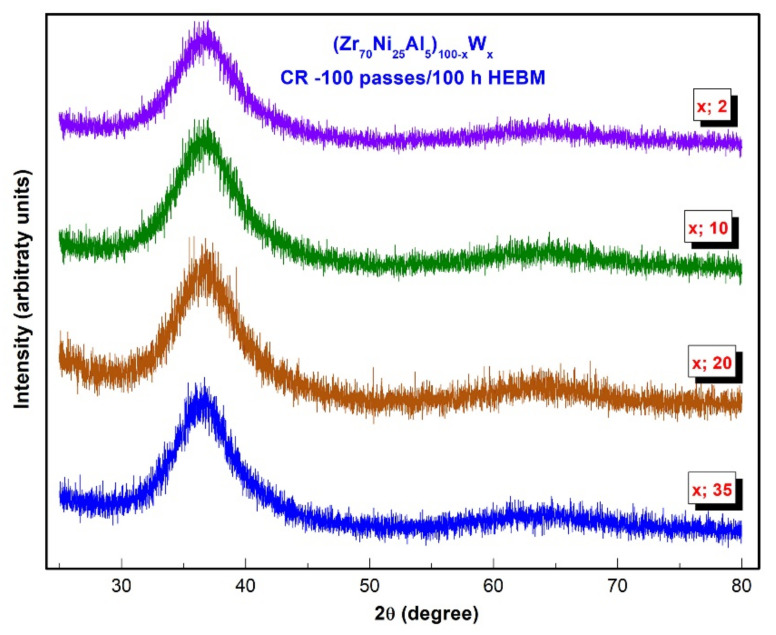
XRD patterns of (Zr_75_Ni_25_Al_5_)_100-x_W_x_ (x; 2, 10, 20, and 35 at. %) powders obtained after 100 passes of CR and then milled for 100 h.

The XRD patterns for the final products of CR 100 passes (Zr_70_Ni_25_Al_5_)_100-x_W_x_ (x; 2, 10, 20, and 35 at. %) obtained after HEBM for 100 h are displayed together in Figure 7. All samples exhibited diffuse halo peaks in the absence of crystalline peaks, indicating the formation of pure amorphous alloy powders.

### 3.2. Thermal Stability

**Figure 8:**
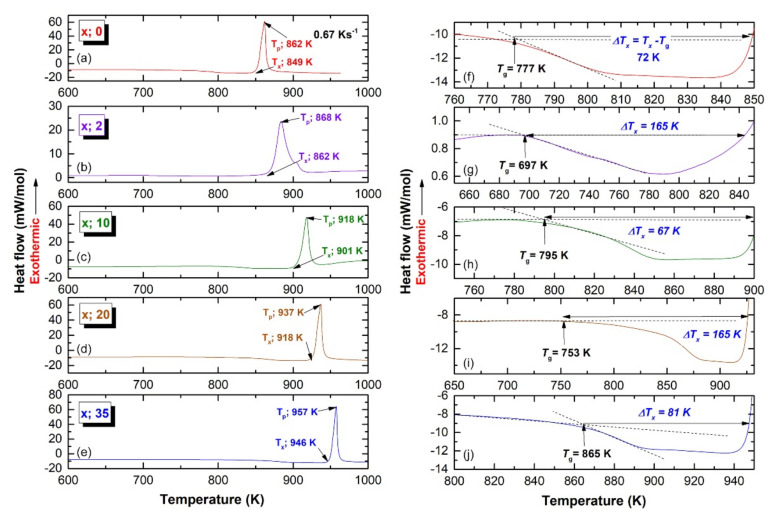
Differential scanning calorimetry (DSC) traces conducted by differential scanning calorimetry (DSC) under He gas flow at a heating rate of 0.67 Ks^−1^ for metallic glassy (Zr_75_Ni_25_Al_5_)_100-x_W_x_ powders milled for 100 h with W concentrations of (**a**) 0, (**b**) 2, (**c**) 10, (**d**) 20, and (**e**) 35 at. %. The supercooled liquid region (ΔT_x_) related to (**a**–**e**) is displayed with a different scale in (**f**–**j**). The glass transition temperature (T_g_), onset-crystallization (T_x_), and peak-(T_p_) temperatures, as well as ΔT_x_, are indexed in the figure by arrow symbols.

**Figure 9:**
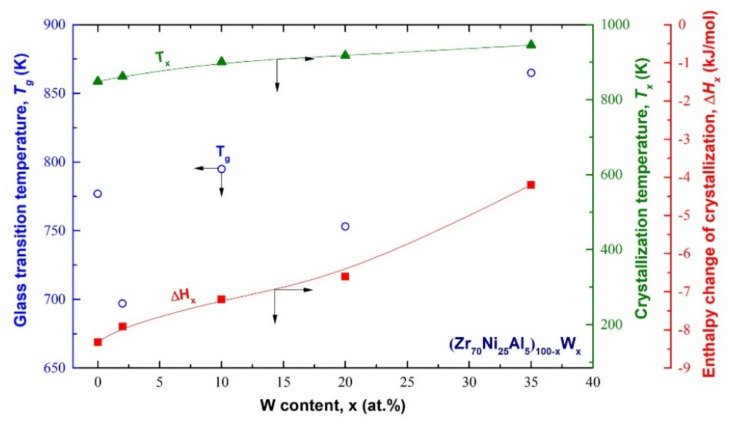
Effect of W (x) concentration on the glass transition (T_g_), crystallization (T_x_) temperatures, supercooled liquid region (ΔT_x_), and enthalpy change of crystallization (ΔH_x_) for (Zr_70_Ni_25_Al_5_)_100-x_W_x_ metallic glassy systems.

The thermal stability for the end product (CR 100 passes/100 h HEBM) of (Zr_70_Ni_25_Al_5_)_100-x_W_x_ (x; 0, 2, 10, 20, and 35 at. %) alloy powders were examined by means of differential scanning calorimetry (DSC) at a heating rate of 40 °C/min under a flow of He gas. Figure 8a–e shows the full-range (600–1000 K) DSC thermograms of all prepared samples, whereas Figure 8f–j shows the DSC traces with a different temperature range. The glass forming ability (GFA), as measured by the glass transition temperature (T_g_), supercooled liquid region (ΔT_x_), crystallization temperature (T_x_), and enthalpy change of crystallization (ΔH_x_) is plotted as a function of W content (x) in Figure 9. Without exception, all samples revealed two opposing events, as shown by the endothermic reaction followed by exothermic peaks (Figure 8a–d). The T_g_, and T_x_ of metallic glassy Zr_70_Ni_25_Al_5_ powder were 777 K (Figure 8f), and 849 K (Figure 8a). This ternary system exhibited a rather wide 72 K, as shown in Figure 8f.

It is worth noting that both T_g_ and ΔT_x_ were independent and did not alter in response to changes the W content (x), as displayed in Figure 8 and Figure 9. The widest ΔT_x_ value (165 K) was obtained with the W contents of 2 at. % (Figure 8g), and 20 at. % (Figure 8i). This implies a good GFA of (Zr_70_Ni_25_Al_5_)_98_W_2_ and (Zr_70_Ni_25_Al_5_)_80_W_20_ metallic glassy systems. In contrast to T_g_, T_x_ is a function of the alloying content of the high-melting temperature phase of W, as can be seen from the monotonous increase in T_x_ upon increasing the W content (Figure 9). The maximum T_x_ value (946 K) was measured for (Zr_70_Ni_25_Al_5_)_65_W_35_ metallic glassy alloy powder, as shown in e. For each system, the ΔH_x_ values were extremely sensitive to the heat of formation (ΔH^for^). High W concentrations (20–35 at. %) may result in decreased ΔH^for^ values, resulting in a rise in ΔH_x_ to approximately −6.3 and −3.9 kJ/mol, respectively (Figure 8). These results were much greater than those obtained at low W concentrations (0–10 at. %), which indicated a higher ΔH_x_ in the range of −8.2 to −6.9 kJ/mol (Figure 9).

The XRD of the (Zr_70_Ni_25_Al_5_)_65_W_35_ sample after annealing at 1000 K is presented in Figure S1. The as-crystalized metallic glassy phase transformed into two crystal phases of monoclinic Zr_4_AlNi_2_ and tetragonal-Ni_4_W, as shown in Figure S1.

**Figure 10:**
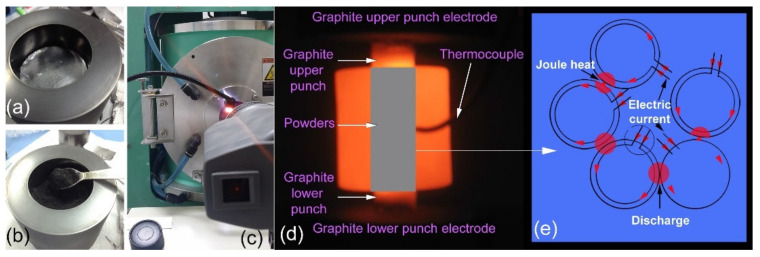
A 50 mm graphite die (**a**) before and (**b**) after charging with metallic glassy (Zr_70_Ni_25_Al_5_)_65_W_35_ powders. The die charged with the powders was mounted inside the SPS unit, where the temperature was monitored by a pyrometer (**c**). A photo of the die during the SPS process is displayed in (**d**). The ON-OFF DC pulsed current route and pulsed current flow via powder particles in the graphite die during the SPS process are shown schematically in (**e**).

Unlike in HP, which requires an external heating source, a pulsed direct current is transmitted through the graphite die and, in some situations, the sample in SPS (Figure 10d). As a result, in SPS, the sample is heated from both the outside and the inside (self-heating). It is worth noting that the sintering temperature can be several hundred degrees lower than what is generally employed in a traditional sintering process, such as HP. SPS is an under-pressure sintering method based on an electrical discharge of pulsed direct current (DC) between the powder particle space and the spark production. The pulsed current propagates over particle surfaces. The local heat created in the discharge column leads in a rapid increase in temperature to more than 910 K when the spark develops into vacant spaces or contact sites of the powder particles. This high temperature causes contaminants to evaporate, as well as evaporation and melting on the surface of powder particles. These melted areas are absorbed toward each other through electron current (ON time) and generating vacuum (OFF time), resulting in the formation of necks between the powder particles. as illustrated in Figure 10e.

### 3.3. Fabrication of Bulk (Zr_70_Ni_25_Al_5_)_100-x_W_x_ Metallic Glassy Systems with the Spark Plasma Sintering (SPS) Technique

Consolidation of temperature-sensitive metastable materials, such as amorphous and metallic glassy alloy powders, is a crucial stage in reproducibly determining the physical and mechanical properties of synthetic materials. For the vast majority of industrial and structural applications, this step is required. Consolidating this class of fine glassy powders into bulk, full-dense compacts while maintaining their original short-range order is a significant challenge. While the majority of metallic glassy powders produced through ball milling may be consolidated using hot pressing (HP) or SPS, SPS has proven to be more advantageous than HP. Some parallels exist between HP and SPS, such as the loading of a powder into a die (Figure 10a,b), the application of external pressure, and the holding of the die during the sintering process (Figure 10c). However, there is a significant difference in the type and the method of heat generation in both strategies.

Besides, when comparing SPS to other conventional sintering techniques, the ON-OFF DC pulse energizing method produces spark plasma, spark impact pressure, joule heating, and an electrical field diffusion effect. Fresh-powder surfaces facilitate the sintering process, as shown by a lower sintering temperature and reduced processing time. Additionally, it has been claimed that joule heating caused by the passage of an electric current through powder particles enhances and improves the welding of the particles when mechanical pressure is applied. Another advantage of SPS is the ability to generate high heating rates during sintering, and sintering of the powder combination may occur in a short processing time. In the present study, the heating rate was in the range between 250 and 300 °C/min.

**Figure 11:**
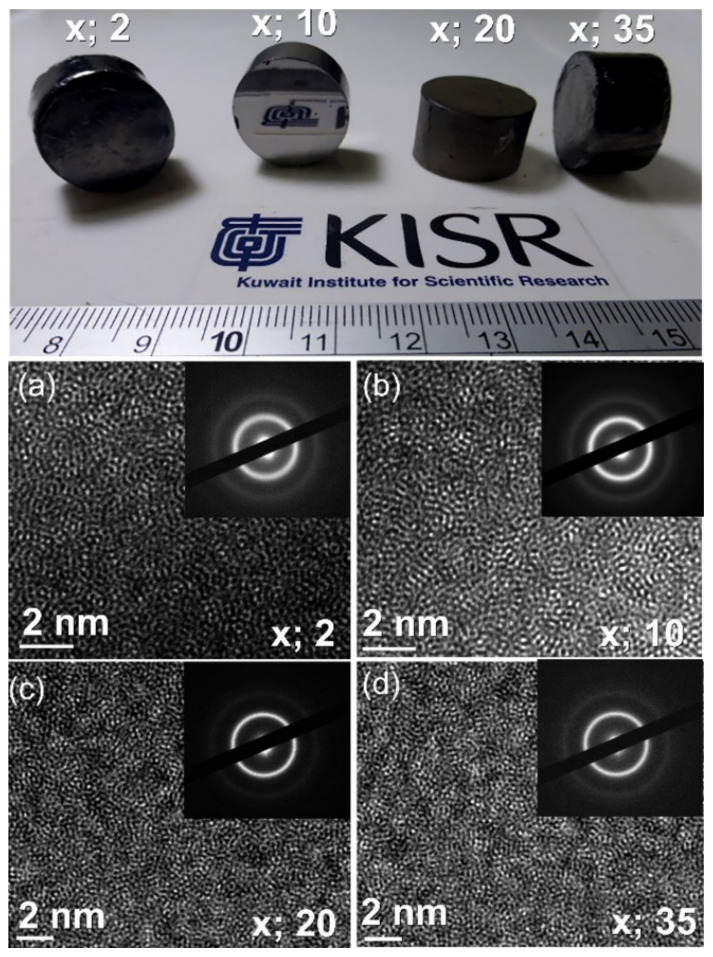
The upper photos display the as-SPS bulk metallic glassy buttons of (Zr_75_Ni_25_Al_5_)_100-x_W_x_ (x; 2, 10, 20, and 35 at. %) alloy powders. The corresponding FE-HRTEM images and related NBDs for x; 2, 10, 20, and 35 at. % are displayed in (**a**–**d**), respectively.

The photographs in Figure 11a displayed as-SPS buttons with a diameter of 20 mm for a selection of (Zr_70_Ni_25_Al_5_)_100-x_W_x_ metallic glassy alloys. These metallic glassy systems exhibited a broad ΔT_x_, ranging from 67 K to 165 K, as shown in Figure 8. The presence of ΔT_x_ enabled the welding of glassy powder particles and provided an excellent opportunity to retain the original short-range order structure after the SPS process. This can be realized from the atomic resolution TEM images and NBDPs for the buttons after SPS (Figure 11a–d). The images revealed maze-like morphology beyond the atomic scale without evidence of the precipitation of crystalline phase. Additionally, the accompanying NBDPs for each image have a characteristic diffuse halo pattern. These results show that without crystallization, the SPS consolidated samples retain their amorphous form.

**Figure 12:**
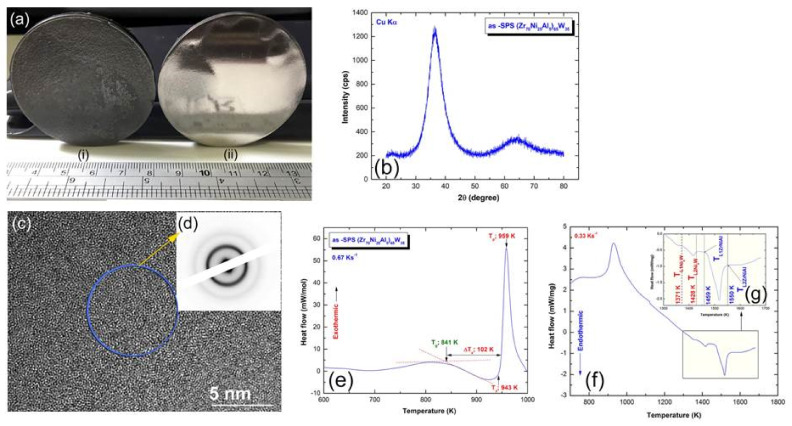
(**a**) Photos of the as-SPS 50 mm (Zr_75_Ni_25_Al_5_)_65_W_35_ metallic glassy system before (**i**) and after (**ii**) polishing. The XRD pattern, HRTEM image with NBDP, DSC thermogram, and DTA trace of the consolidated powders are presented in (**b**), (**c**,**d**), (**e**), and (**f**,**g**), respectively.

To verify the ability of producing an exceptionally large (Zr_70_Ni_25_Al_5_)_65_W_35_ bulk metallic glassy (BMG) alloy, the as-milled glassy powders were charged into a 50 mm graphite die with a high aspect ratio of 10:1 (Figure 10b). The system was then fed into the SPS machine, where it was heated to 920 K at a rate of 300 °C/min for the consolidation process. Figure 12(ai,aii) show the as-SPS 50 mm BMG buttons before and after polishing. The polished glassy button had a smooth surface and glistened metallically (Figure 12(aii)). Furthermore, the consolidated sample exhibited a dense look devoid of fractures or holes.

The general crystal structure of the as-SPS (Zr_70_Ni_25_Al_5_)_65_W_35_ sample was examined by XRD. This bulk sample revealed a broad diffuse smooth halo, suggesting the presence of amorphous phase and the successful completion of the SPS process (Figure 12b). Figure 12c shows the HRTEM image of the consolidated sample in tandem with the NBDP (Figure 12d). No characteristics of locally ordered areas could be identified, and the image depicts an amorphous phase with a homogeneous maze contrast (Figure 12c). The NBDP exhibits the characteristic diffuse halo of an amorphous phase, as presented in Figure 12d. The DSC curve of this BMG sample shows the two opposing reactions (Figure 12e) previously shown for the glassy powders prior to consolidation (Figure 8e,j), with close values of T_g_, T_x_, and ΔT_x_, which are in reasonable agreement with the original metallic glassy powders. The differential thermal analysis (DTA) method was used to determine the melting (T_m_) and liquidus (T_l_) temperatures of the as-SPS (Zr_70_Ni_25_Al_5_)_65_W_35_ sample. Figure 12f shows the full-scale DTA curve, where Figure 12g displays the DTA thermogram in a temperature range between 1200 and 1700 K. The DTA curve and corresponding XRD analysis indicated that the melting process took place through two stages. In the first stage that was performed at 1371 K, the Ni_4_W phase was yielded, where the second endothermic peak (1459 K) was related to the melting of the Zr_4_AlNi_2_ phase.

**Figure 13:**
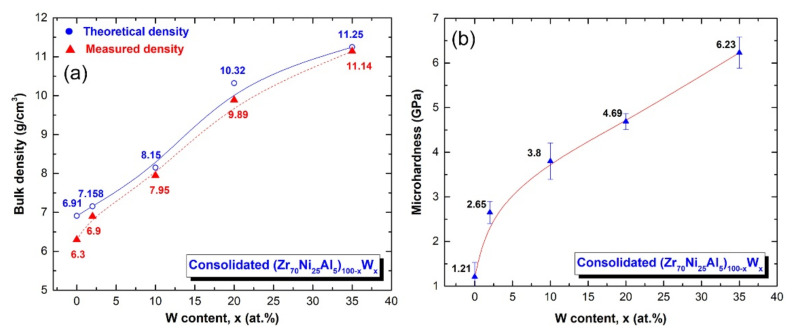
Effect of W (x) concentration on the (**a**) bulk density and (**b**) microhardness of (Zr_70_Ni_25_Al_5_)_100-x_W_x_ metallic glassy systems.

The bulk density and Vickers microhardness as a function of W (x) content are shown in Figure 13a,b, respectively. In both figures, the displayed points represent the average value of 10 separate measurements. The observed densities were shown with the estimated densities for each composition in Figure 13a. In general, the bulk density values of (Zr_70_Ni_25_Al_5_)_100-x_W_x_ BMG systems increase monotonically as the W content increases. The bulk density readings, which varied from 6.3 to 11.14 g/cm^3^, were quite consistent with the predicted value, as shown in Figure 13a.

Vickers microhardness measurements on (Zr_70_Ni_25_Al_5_)_100-x_W_x_ BMG with a 500 g load reveal a roughly linear relationship between hardness and W concentration, as shown in Figure 13b. It can be seen that increasing the W content from 0 to 35 at. % resulted in a substantial increase in microhardness from 1.2 to 6.2 GPa, as shown in Figure 13b.

## 4. Conclusions

Novel metallic glassy (Zr_70_Ni_25_Al_5_)_100-x_W_x_ (x; 0, 2, 5, 10, 20, 35 at. %) powders were prepared by high-energy ball milling the cold rolled elemental powders for 100 h. Mechanical treatment of the powders utilizing a two-drum conventional cold rolling machine for 100 times prior to milling was a critical step in obtaining primary homogeneous feedstock materials in this research. After 50 passes, a nanocrystalline bcc-W(ZrAlNi) solid-solution was produced due to plastic deformation and lattice defects introduced into the cold-rolled powders. As shown by the substantial increase in the lattice parameter, further passes (100 times) resulted in additional expansion of the solid solution lattice. When cold-rolled powders were processed in a high-energy ball mill, several kinds of lattice defects were introduced, including dislocations and point defects. Due to the fact that bcc-solid solution is a metastable phase, it tended to transform monotonically into another metastable phase (amorphous) when the ball milling duration was increased (25–50 h). After 100 passes of milling, the bcc-solid solution fully transformed into the amorphous phase. The current study established the impact of W alloying on the thermal stability of the metallic glass phase by increasing the crystallization temperature from 849 K (x; 0 at. %) to 946 K (x; 35 at. %). The glass transition temperatures and supercooled liquid regions of these systems, on the other hand, are not dependent on the W concentration. It should be noted that the broad supercooled liquid regions of these systems, ranging from 67 to 165 K, afforded an ideal opportunity for effective powder consolidation into large bulk metallic glasses (20 mm to 50 mm) without crystallization using the spark plasma sintering technique. The density measured for as-consolidated buttons increased with the increase of the W content from 6.3 g/cm^3^ (x; 0 at. %) to 11.14 g/cm^3^ (x; 35 at. %), which is in fair accord with the theoretical values. Likewise with density, increasing the W concentration led to a significant increase in the microhardness, which tended to increase from 1.21 GPa (x; 0) to 6.23 GPa (x; 35 at. %).

In our near future plan, the bulk metallic glassy (Zr_70_Ni_25_Al_5_)_100-x_W_x_ systems will be characterized using the nanoindentation technique in order to estimate their modulus of elasticity (E), poison’s ratio, and nanohardness. The indents created by the nanoindentation test will be studied using an atomic force microscope (AFM) to obtain a better understanding of the glassy system’s response to plastic deformation. Additionally, the metallic glassy powders will be employed to coat steel substrates utilizing the cold spray technique. Corrosion and wear characteristics of the metallic glassy protective layer will be determined.

## Figures and Tables

**Table 1:** Local elemental EDS analysis (at. %) of (Zr_70_Ni_25_Al_5_)_65_W_35_ powders obtained after 12.5 h of ball milling and 100 passes of cold rolling.

	12.5 h							100 Passes CR							
**Element**	**A**	**B**	**C**	**D**	**E**	**H**	**G**	**I**	**II**	**III**	**IV**	**V**	**VI**	**VII**	**VIII**
Zr	2.5	3.7	8.7	2.1	4.2	1.8	36.4	44.8	46.1	42.5	41.6	42.8	48.3	45.2	44.2
Ni	1.0	92.8	83.0	1.9	2.1	3.6	24.7	17.2	15.3	18.7	14.3	15.1	14.8	16.1	18.3
Al	96.1	1.6	2.1	95.2	92.6	1.2	32.8	2.9	3.4	2.8	3.2	2.9	3.6	3.1	2.8
W	0.3	1.8	6.1	0.7	1.0	93.3	6.0	35.1	35.2	36.0	40.9	39.2	33.3	35.6	37.7
Fe	0.1	0.1	0.1	0.1	0.1	0.1	0.1	-	-	-	-	-	-	-	-

## Data Availability

Not available.

## Supplementary Materials

The following are available online at https://www.mdpi.com/article/10.3390/nano11112952/s1, Figure S1: The XRD of the (Zr_70_Ni_25_Al_5_)_65_W_35_ sample after annealing at 1000 K.

## References

[1] Klement W., Willens R.H., Duwez P. (1960). Non-crystalline structure in solidified gold–silicon alloys. Nature.

[2] Cheng Y.T., Johnson W.L., Nicolet M.A. (1988). Crystal-to-glass transformation in metallic materials. Mater. Sci. Eng..

[3] Duwez P., Willens R.H., Klement W. (1960). Continuous Series of Metastable Solid Solutions in Silver-Copper Alloys. J. Appl. Phys..

[4] Lord Rayleigh O.M.F.R.S. (2009). On convection currents in a horizontal layer of fluid, when the higher temperature is on the under side. Phil. Mag..

[5] Herd S.R., Tu K.N., Ahn K.Y. (1983). Formation of an amorphous Rh-Si alloy by interfacial reaction between amorphous Si and crystalline Rh thin films. Appl. Phys. Lett..

[6] Blatter A., Allmen M. (1985). Reversible amorphization in laser-quenched titanium alloys. Phys. Rev. Lett..

[7] Yeh X.L., Samwer K., Johnson W.L. (1983). Formation of an amorphous metallic hydride by reaction of hydrogen with crystalline intermetallic compounds—A new method of synthesizing metallic glasses. Appl. Phys. Lett..

[8] Aoki K., Nagano M., Yanagitani A., Masumoto T. (1987). Hydrogen-induced amorphization and its effect on magnetic properties of the Laves-phase GdFe_2_ compound. J. Appl. Phys..

[9] Taguchi K., Shinozaki K., Okumura H., Michioka C., Yoshimura K., Ishihara K.N. (2020). Discovery of Amorphous Iron Hydrides via Novel Quiescent Reaction in Aqueous Solution. Sci. Rep..

[10] Schwarz R.B., Johnson W.L. (1983). Formation of an Amorphous Alloy by Solid-State Reaction of the Pure Polycrystalline Metals. Phys. Rev. Lett..

[11] Barbour J.C., Nastasi M., Mayer J.W. (1986). Mobility of Ni versus Zr in an amorphous Ni-Zr alloy. Appl. Phys. Lett..

[12] Shi H., Zhao W., Wei X., Ding Y., Shen X., Liu W. (2019). Effect of Ti addition on mechanical properties and corrosion resistance of Ni-free Zr-based bulk metallic glasses for potential biomedical applications. J. Alloy. Compd..

[13] Han K., Wang Y., Qiang J., Jiang H., Gu L. (2019). Low-cost Zr-based bulk metallic glasses for biomedical devices applications. J. Non Cryst. Solids.

[14] El-Eskandrany M.S., Al-Azmi A. (2016). Potential applications of cold sprayed Cu_50_Ti_20_Ni_30_ metallic glassy alloy powders for antibacterial protective coating in medical and food sectors. J. Mech. Behav. Biomed. Mater..

[15] Han K., Qiang J., Wang Y., Häussler P. (2017). Zr-Al-Co-Cu bulk metallic glasses for biomedical devices applications. J. Alloy. Compd..

[16] Rajan T., Arockiarajan A. (2021). Thin film metallic glasses for bioimplants and surgical tools: A review. J. Alloy. Compd..

[17] Pan J., Ivanov Y.P., Zhou W.H., Li Y., Greer A.L. (2020). Strain-hardening and suppression of shear-banding in rejuvenated metallic glass. Nature.

[18] Greer A.L. (1993). Confusion by Design. Nature.

[19] Inoue A. (1995). High strength bulk amorphous alloys with low critical cooling rates (overview). Mater. Trans. JIM.

[20] Bajpai S., Nisar A., Sharma R.K., Schwarz U.D., Balani K., Datye A. (2021). Effect of fictive temperature on tribological properties of Zr_44_Ti_11_Cu_10_Ni_10_Be_25_ bulk metallic glasses. Wear.

[21] Yang W., Sun X., Liu H., Yu C., Li W., Inoue A., Şopu D., Eckert J., Tang C. (2020). Structural homology of the strength for metallic glasses. J. Mater. Sci. Technol..

[22] Tan Y., Wang Y.W., Cheng X.W., Fu Q., Xin Z.H., Xu Z.Q., Cheng H.W. (2021). Effects of Al replacement on glass forming ability and mechanical properties of Zr-based bulk metallic glasses. J. Non Cryst. Solids.

[23] Polosan S., Ganea P., Nitescu A. (2021). Structural, magneto-optical and dielectric properties of phosphate tellurite glasses. Mater. Res. Bull..

[24] Chen S.H., Yue T.M., Tsui C.P., Chan K.C. (2016). Flaw-induced plastic-flow dynamics in bulk metallic glasses under tension. Sci. Rep..

[25] Li W., Gao Y., Bei H. (2016). Instability analysis and free volume simulations of shear band directions and arrangements in notched metallic glasses. Sci. Rep..

[26] Herrero-Gómez C., Samwer K. (2016). Stress and temperature dependence of the avalanche dynamics during creep deformation of metallic glasses. Sci. Rep..

[27] Chen C., Gao M., Wang C., Wang W.-H., Wang T.-C. (2016). Fracture behaviors under pure shear loading in bulk metallic glasses. Sci. Rep..

[28] Yang W., Liu H., Zhao Y., Inoue A., Jiang K., Huo J., Ling H., Li Q., Shen B. (2014). Mechanical properties and structural features of novel Fe-based bulk metallic glasses with unprecedented plasticity. Sci. Rep..

[29] Hitit A., Şahin H. (2017). The effect of iron content on glass forming ability and thermal stability of Co–Fe–Ni–Ta–Nb–B–Si bulk metallic glass. Metals.

[30] Shi Z., Li R., Li X., Wang C., Zhang T. (2019). Controllable brittleness in soft-magnetic Fe-P-C-B metallic glasses through composition design. Mater. Sci. Eng. A.

[31] Xu K., Ling H., Li Q., Li J., Yao K., Guo S. (2014). Effects of Co substitution for Fe on the glass forming ability and properties of Fe_80_P_13_C_7_ bulk metallic glasses. Intermetallics.

[32] Xu S., Wang J., Wang N., Han Z.T., Wan Y. (2021). Soft magnetic properties and corrosion resistance of the annealed (Fe_0.7_Co_0.15_Ni_0.15_)_75_B_21_Nb_4_ metallic glasses. Mater. Today Commun..

[33] Cui G., Li X., Shan G., Gao H., Wong K.W., Zhang J. (2020). Depression of direct exchange couplings in metallic glasses: A comparative study of critical and electronic behavior in Gd_6_Co_4.85_ intermetallic compound and metallic glass. Intermetallics.

[34] Chen H., Dong B., Zhou S., Li X., Qin J. (2018). Structural, magnetic, and electronic properties of Fe_82_Si_4_B_10_P_4_ metallic glass. Sci. Rep..

[35] Zhang J., Shan G., Li J., Wang Y., Shek C.H. (2021). Structures and physical properties of two magnetic Fe-based metallic glasses. J. Alloy. Compd..

[36] Ayyagari A., Hasannaeimi V., Arora H., Mukherjee S. (2018). Electrochemical and Friction Characteristics of Metallic Glass Composites at the Microstructural Length-scales. Sci. Rep..

[37] Burkov A., Chigrin P. (2018). Effect of tungsten, molybdenum, nickel and cobalt on the corrosion and wear performance of Fe-based metallic glass coatings. Surf. Coat. Technol..

[38] Si J.J., Chen X.H., Cai Y.H., Wu Y.D., Wang T., Hui X.H. (2016). Corrosion behavior of Cr-based bulk metallic glasses in hydrochloric acid solutions. Corros. Sci..

[39] El-Eskandarany M. (2016). Metallic glassy Zr_70_Ni_20_Pd_10_ powders for improving the hydrogenation/dehydrogenation behavior of MgH_2_. Sci. Rep..

[40] El-Eskandarany M.S., Al-Salem S.M., Ali N., Banyan M., Al-Ajmi F., Al-Duweesh A. (2020). From gangue to the fuel-cells application. Sci. Rep..

[41] El-Eskandarany M.S. (2019). Recent developments in the fabrication, characterization and implementation of MgH_2_-based solid-hydrogen materials in the Kuwait Institute for Scientific Research. RSC Adv..

[42] El-Eskandarany M.S. (2019). Metallic glassy Ti_2_Ni grain-growth inhibitor powder for enhancing the hydrogenation/dehydrogenation kinetics of MgH_2_. RSC Adv..

[43] Zhao B., Zeng S., Qin X., Li Z., Zhang S., Zhang H., Zhu Z. (2021). Cu-Ag nanocomposites in-situ formed on the surface of CuZr-based metallic glasses as highly efficient catalysts for improving catalytic behavior and reusability towards wastewater treatment. Appl. Surf. Sci..

[44] Hattori M., Katsuragawa N., Yamaura S., Ozawa M. (2021). Three-way catalytic properties and microstructures of metallic glass driven composite catalysts. Catal. Today.

[45] Zhao B., Zhu Z., Dong X.Q., Li Z., Zhang H. (2020). Highly efficient and stable CuZr-based metallic glassy catalysts for azo dye degradation. J. Mater. Sci. Technol..

[46] Hasannaeimi V., Mukherjee S. (2019). Noble-metal based metallic glasses as highly catalytic materials for hydrogen oxidation reaction in fuel cells. Sci. Rep..

[47] Chen Y., Dai Z.W., Jiang J.Z. (2021). High entropy metallic glasses: Glass formation, crystallization and properties. J. Alloy. Compd..

[48] Suryanarayana C., Inoue A. (2018). Bulk Metallic Glasses.

[49] Sun Y., Concustell A., Greer A. (2016). Thermomechanical processing of metallic glasses: Extending the range of the glassy state. Nat Rev. Mater..

[50] Axinte E. (2012). Metallic glasses from “alchemy” to pure science: Present and future of design, processing and applications of glassy metals. Mater. Des..

[51] Chen M. (2011). A brief overview of bulk metallic glasses. NPG Asia Mater..

[52] Greer A.L. (2009). Metallic glasses on the threshold. Mater. Today.

[53] Inoue A., Takeuchi A. (2011). Recent development and application products of bulk glassy alloys. Acta Mater..

[54] Johnson W.L. (1999). Bulk glass-forming metallic alloys: Science and technology. MRS Bull..

[55] Koch C.C., Cavin O.B., McKamey C.G., Scarbrough J.O. (1983). Preparation of “amorphous” Ni_60_Nb_40_ by mechanical alloying. Appl. Phys. Lett..

[56] El-Eskandarany M.S. (2020). Mechanical Alloying: Energy, Surface Protective Coating and Medical Applications.

[57] Battezzati L., Enzo S., Schiffini L., Cocco G. (1988). Formation and crystallization of amorphous Ni_2_Ti powders prepared by mechanical alloying. J. Less Common Met..

[58] Weeber A.W., Loeff P.I., Barker H. (1988). Glass-forming range of transition metal-transition metal alloys prepared by mechanical alloying. J. Less Common Met..

[59] Schultz L. (1988). Glass-forming ranges in transition metal-Zr alloys prepared by mechanical alloying. J. Less Common Met..

[60] Hellstern E., Schultz L. (1988). Progress of the amorphization reaction during mechanical alloying in Fe-Zr. J. Appl. Phys..

[61] El-Eskandarany M.S., Itoh F., Aoki K., Suzuki K. (1990). Preparation of Al_x_Ta_1-x_ amorphous alloy powder by mechanical alloying. J. Non Cryst. Solids.

[62] El-Eskandarany M.S., Aoki K., Suzuki K. (1991). Calorimetric characterization of the amorphization process for milled Al_50_Nb_50_ alloy powders. Scr. Metall. Mater..

[63] El-Eskandarany M.S. (1999). Solid-state amorphization reaction in mechanically deformed Al_x_Hf_100−x_ multilayered composite powders and the effect of annealing. J. Alloy. Compd..

[64] Fukunaga T., Nakamura K., Suzuki K., Mizutani U. (1990). Amorphization of immiscible CuTa system by mechanical alloying and its structure observation. J. Non Cryst. Solids.

[65] El-Eskandarany M.S., Matsushita M., Inoue A. (2001). Phase transformations of ball-milled Nb_50_Zr_10_Al_10_Ni_10_Cu_20_ powders and the effect of annealing. J. Alloy. Comp..

[66] El-Eskandarany M.S., Inoue A. (2002). New V_45_Zr_20_Ni_20_Cu_10_Al_2.5_Pd_2.5_ glassy alloy powder with wide supercooled liquid region. Mater. Trans. JIM.

[67] El-Eskandarany M.S., Zhang W., Inoue A. (2003). Mechanically induced crystalline-glassy phase transformations of mechanically alloyed Ta_55_Zr_10_Ni_10_Al_10_Cu_15_ multicomponent alloy powders. J. Alloy. Compd..

[68] El-Eskandarany M.S., Saida J., Inoue A. (2003). Mechanically induced solid state devitrifications of glassy Zr_65_Al_7.5_Ni_10_Cu_12.5_Pd_5_ alloy powders. Acta Mater..

[69] El-Eskandarany M.S., Ishihara S., Inoue A. (2003). Mechanism of solid-state reaction for fabrication of new glassy V_45_Zr_22_Ni_22_Cu_11_ alloy powders and subsequent consolidation. J. Mater. Res..

[70] El-Eskandarany M.S., Inoue A. (2006). Synthesis of new bulk metallic glassy Ti_60_Al_15_Cu_10_W_10_Ni_5_ alloy by hot-pressing the mechanically alloyed powders at the supercooled liquid region. Met. Trans. A.

[71] El-Eskandarany M.S., Ishihara S., Inoue A. (2005). Fabrication and characterizations of new glassy Co_71_Ti_24_B_5_ alloy powders and subsequent hot pressing into a fully dense bulk glass. Met. Trans. A.

[72] Madge S.V., Caron A., Gralla R., Wilde G., Mishra S.K. (2014). Novel W-based metallic glass with high hardness and wear resistance. Intermetallics.

[73] El-Eskandarany M.S., Sumiyama K., Suzuki K. (1997). Crystalline-to-amorphous phase transformation in mechanically alloyed Fe_50_W_50_ powders. Acta Metall..

[74] Kotynia K., Pawlik P., Filipecka K., Filipecki J. (2020). Calorimetric and structural analysis of the Zr-;Fe-Co-B-Mo-W amorphous alloys doped with gadolinium. J. Alloy. Compd..

[75] El-Eskandarany M.S., Al-Hazza A., Al-Hajji L.A., Ali N., Al-Duweesh A.A., Banyan M., Al-Ajmi F. (2021). Mechanical Milling: A Superior Nanotechnological Tool for Fabrication of Nanocrystalline and Nanocomposite Materials. Nanomaterials.

