# Supplementary material for: Phase Transformations from Nanocrystalline to Amorphous (Zr70Ni25Al5)100-xWx (x; 0, 2, 10, 20, 35 at. %) and Subsequent Consolidation
Source: Nanomaterials (Basel). 2021 Nov 3;11(11):2952. doi: 10.3390/nano11112952 (PMC8618145; doi:10.3390/nano11112952)
Supplement: Supplementary file 1 [file nanomaterials-11-02952-s001.zip › nanomaterials-1427127-supplementary.pdf]

Supplementary Materials

Phase Transformations from Nanocrystalline to Amorphous $(Zr_{70}Ni_{25}Al_5)_{100-x}W_x$ (x; 0, 2, 10, 20, 35 at. %) and Subsequent Consolidation

M. Sherif El-Eskandarany *, Naser Ali, Fahad Al-Ajmi † and Mohammad Banyan †

[1] Nanotechnology and Applications Program, Energy and Building Research Center, Kuwait Institute for Scientific Research, Safat 13109, Kuwait; nmali@kisr.edu.kw (N.A.); ftajmi@kisr.edu.kw (F.A.-A.); mbanyan@kisr.edu.kw (M.B.)
* Correspondence: msherif@kisr.edu.kw
† F.A.-A and M.B. contributed equally to this work.

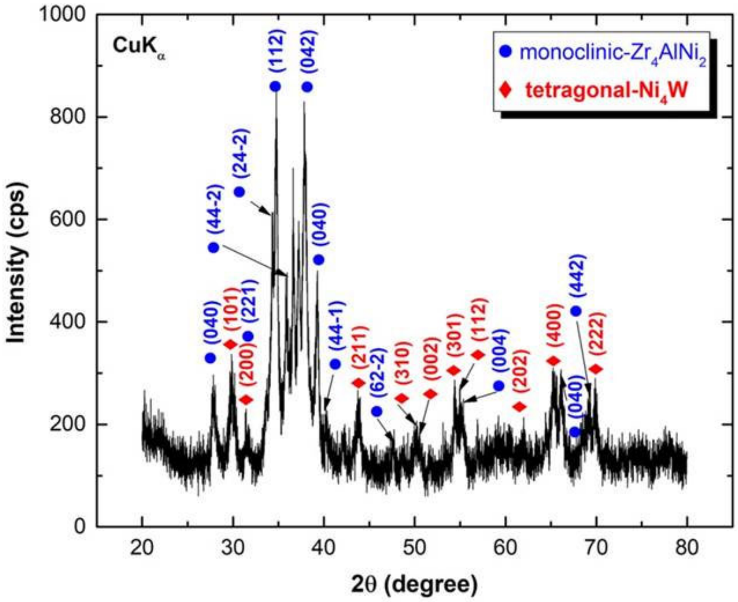

Figure S1. The XRD of the $(Zr_{70}Ni_{25}Al_5)_{65}W_{35}$ sample after annealing at 1000 K.
